# Genetics of selenoproteins and selenoprotein metabolism – An overview of current concepts and emerging aspects

**DOI:** 10.1016/j.redox.2026.104083

**Published:** 2026-02-12

**Authors:** Ulrich Schweizer, Marietta Fabiano

**Affiliations:** Universität Bonn, Universitätsklinikum Bonn, Institut für Biochemie und Molekularbiologie, Bonn, 53113, Germany

**Keywords:** Selenium, Ferroptosis, Mutation, Inborn, Human

## Abstract

Selenoproteins are proteins containing the 21st proteinogenic amino acid selenocysteine in their peptide chain. Selenocysteine lends specific properties to selenoproteins, and selenoenzymes are often catalytically superior to enzymes that would contain cysteine instead. Degradation of peroxides, in particular by GPX4, the master regulator of ferroptosis, is currently receiving a lot of attention owing to its potential in cancer or degenerative disorders. In this context, selenium metabolism has recently become again an active field of research based on the importance to provide selenium to GPX4. Apart from redox control, deiodinases are being identified as critical modulators of an increasing number of specific developmental decisions in many organs where they are modulating local thyroid hormone levels. An entirely new field of selenium research is centered around the biosynthesis of ethanolamine phospholipids. Overall, skeletal muscle, the endocrine system, the immune system, and in particular the brain, are sensitive to selenoprotein deficiency. We summarize the basic concepts of selenium and selenoprotein metabolism and provide a framework where selenium or selenoproteins are important for human health. Then we review new work that significantly advanced our understanding of selenium biology in humans and summarize the substantial body of data describing patients with inborn errors of selenoprotein biosynthesis or selenoprotein genes. Transgenic mouse models are reported where they help define a biological concept. We deliberately did not strive to cover in this work all the biochemical mechanisms of selenoenzymes or their interactions with effector proteins.

## Standing on the shoulders of giants

Before summarizing the underlying concepts and recent advances that have expanded our understanding of selenoproteins in mammalian physiology and human disease, it is worth acknowledging the pioneering scientist who first identified selenium as an essential trace element in mammals.

The discovery originated from nutritional studies in animal models — notably rats and chickens — that were fed semisynthetic diets. These regimens induced “dietary liver degeneration” in rats and “exudative diathesis” in chickens. Remarkably, three dietary factors could prevent these pathologies: (i) cysteine or other sulfur-containing amino acids (at relatively high doses), (ii) α-tocopherol (vitamin E), and (iii) an enigmatic factor then referred to as “factor 3.”

The year 2026 marks, in a sense, mark the 75th anniversary of the critical discovery that ultimately led to the identification of selenium as this “factor 3.” While most researchers in the field point to Klaus Schwarz's landmark 1957 paper in The Journal of the American Chemical Society [1] as the defining moment, his earlier works from 1951 to 1954 are equally important. In these studies, Schwarz described the puzzling observation that European yeast used as a protein source in a semisynthetic rat diet caused dietary liver degeneration in the context of vitamin E deficiency, whereas American yeast did not [2,3].

The explanation is now clear for us: the growth substrates for yeast fermentation differed between Europe and North America, leading to differences in selenium content within the protein fraction from the yeast. This realization emerged from a frustrating period in which Schwarz's central animal model failed after his translocation from the Kaiser Wilhelm Institute in Heidelberg, Germany, to the National Institutes of Health in Bethesda, MD, USA.

It would take several more years to chemically purify “factor 3” and show it to contain selenium [1]. Interestingly, back in Heidelberg, Schwarz had also demonstrated that vitamin E could prevent dietary liver degeneration [4]. As we will see later, this early link between selenium and vitamin E remains relevant today, and even explains the supportive role of sulfur-containing amino acids.

The other pioneer that should be mentioned at this point is August Böck, whose laboratory in Munich elucidated the whole selenoprotein biosynthetic pathway using genetics and molecular biology in *E. coli* [5]. It is on the basis of his seminal biochemical and genetic work on bacterial selenoprotein biosynthesis that the components and pathways in eukaryotes have been delineated.

## 1 Seminal observations on organ systems affected by low selenium or by selenoprotein deficiency

It is beyond the scope of this review to comprehensively describe all symptoms and syndromes that have been associated with selenium (Se) deficiency in humans and livestock since the seminal work of Klaus Schwarz. However, recent advances in genetics now address several longstanding questions in the field, and these historical observations provide essential context for interpreting recent findings [6]. In order to simplify nomenclature, this work makes exclusive use of the standardized nomenclature on selenoproteins, even if the cited work used older abbreviations [7].

### 1.1 Skeletal muscle, heart, and vascular system

In poultry, combined selenium and vitamin E deficiency causes hemorrhagic diathesis, a vascular disorder characterized by increased capillary permeability, damage to blood vessel walls, and subcutaneous hemorrhage [8]. In many mammalian livestock species, severe dietary deficiency of selenium (often together with vitamin E) leads to nutritional myopathy or white muscle disease [9], affecting both skeletal and cardiac muscle.

In humans, Keshan disease — an endemic cardiomyopathy first described in rural China — is associated with low selenium status [10,11]. Similar pathology has been observed in patients receiving long-term selenium-deficient total parenteral nutrition [[12], [13], [14]].

The first genetic disorder linked to deficiency of a specific selenoprotein was SELENON-related myopathy [15,16]. Although the precise biochemical function of SELENON remains under investigation, it is clear that pathogenic mutations affecting the incorporation of selenocysteine (Sec) into the protein, account for the malfunction of the protein, even if the rest of the polypeptide remains intact [17,18].

### 1.2 Blood and immune system

Keshan disease may be exacerbated by infection with certain Coxsackie viruses, which become more virulent under selenium-deficient conditions [19,20]. Animal experiments show that tissue damage is generally more severe when selenium status is low or when one of the most abundant selenoproteins, glutathione peroxidase 1 (GPX1), is genetically inactivated. Also, influenza virus evolves to greater pathogenicity in *Gpx1*-deficient mice [21]. Interestingly, selenophosphate synthase, a key enzyme in selenoprotein biosynthesis, was initially cloned as a gene induced in activated CD4^+^ T-cells [22].

Cellular GPX was already known as an enzyme highly efficient in the glutathione (GSH)-dependent reduction of hydrogen peroxide. However, it was only within the context that selenium deficiency induced hemolysis in rats, that GPX was recognized as the first mammalian selenoenzyme [23]. Around the same time, Flohé et al. demonstrated that GPX isolated from human erythrocytes contains exactly one atom of selenium per protein molecule [24]. Because GPX1 expression is highly responsive to selenium availability [25], it became widely accepted that “selenium is a potent antioxidant”, a concept that has shaped decades of research.

### 1.3 Thyroid hormone metabolism

The second mammalian selenoenzyme to be identified was Type I iodothyronine deiodinase (DIO1). In 1990, three independent research groups demonstrated that DIO1 is a selenoenzyme [26,27] and cloned its cDNA, again revealing a UGA codon for selenocysteine within the open reading frame [28]. Years before it was a puzzling finding that in the *Gpx1* gene a UGA/stop codon was at the position where Sec should be coded [29].

**Fig. 1:**
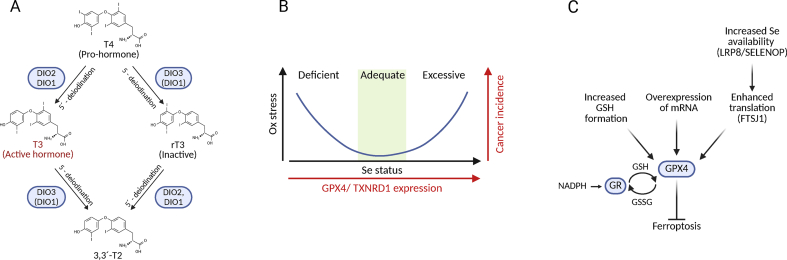
Key concepts of selenoprotein actions. A) Deiodinases (DIO1-3) can locally activate and inactivate thyroid hormones. 3,3′,5,5′-tetraiodothyronine (T4) is the major product of the thyroid gland, but represents a pro-hormone. 5′-deiodination through DIO1 or DIO2 generates 3,3′,5-triiodothyronine (T3), the receptor-binding hormone. 5-deiodination through DIO1 or DIO3 results in the generation of the inactive 3,5,5′-triiodothyronine (rT3) or 3,3′-T2 from T4 or T3, respectively. B) Oxidative stress and cancer incidence increase both under conditions of low selenium and as a result of excessive selenium status. Activities of selenoenzymes GPX4 and TXNRD1 correlate with selenium status. At low selenium status oxidative stress may favor mutation and tumor transformation, while at high GPX4 and TXNRD1 expression cells destined for ferroptosis will be relatively protected from cell death. C) GPX4 is the key regulator of ferroptosis. This activity of the enzyme depends on the availability of glutathione (GSH), which is reduced by glutathione reductase (GR), on the expression level of its mRNA, on the efficiency of translation of selenoproteins, and on the uptake and availability of selenium for the biosynthesis of selenocysteine, e.g. through SELENOP and its endocytic receptor, LRP8.

The thyroid gland produces primarily thyroxine (3,3′,5,5′-tetraiodothyronine, T4), an inactive prohormone (Fig. 1A). The biologically active hormone is 3,3′,5- triiodothyronine (T3), which binds to nuclear T3 receptors. Conversion of T4 to T3 requires 5′-deiodinase activity, catalyzed by DIO1 or the later discovered DIO2. Conversely, DIO3 inactivates T4 and T3 by converting them into reverse T3 (rT3) and 3,3′-diiodothyronine (T2), respectively. Thus, selenoenzymes of the deiodinase family are critical regulators of thyroid hormone action in a tissue- and stage-specific manner [[30], [31], [32]]. Interestingly, also developmental decisions, e.g. muscle stem cell proliferation and lineage progression maturation of inner ear and retina, are regulated by expression of DIO2 and DIO3 [30,[32], [33], [34]]. Given the central roles of thyroid hormones in the development of many organs and in the regulation of metabolism, impaired selenium availability may impact many organ systems [35].

### 1.4 Cancer and ferroptosis

The Nutritional Prevention of Cancer (NPC) Trial was a highly influential study in the field. It was a randomized, double-blind, placebo-controlled study in over 1300 men with a history of skin cancer and investigated the effects of selenium supplementation over a mean period of 7.7 years in >1.300 male probands [36]. The study reported a 25% reduction in overall cancer incidence and a 63% reduction in prostate cancer incidence, confirmed after a further 7 years of follow-up.

Historically, the proposed protective effect of selenium against cancer was linked to its antioxidant function. Because early human selenoproteins were identified as glutathione peroxidases, which reduce reactive oxygen species, low selenium was thought to weaken DNA protection and increase mutational load (Fig. 1B). Supporting this, a GPX1 polymorphism (Pro198Leu, rs1050450) associated with lower enzyme activity has been linked to increased cancer risk [37]. In line with this concept, the risk for prostate cancer correlated negatively with selenium status in carriers of the high-risk allele [38].

The U-shaped relationship between selenium status and cancer incidence may reflect two mechanisms (Fig. 1B). At low selenium levels, antioxidant defense is compromised and mutational load increases; at high selenium levels, some tumors may benefit from overexpression of certain selenoenzymes. For example, the third selenoenzyme (family) discovered in human cells was thioredoxin reductase (TXNRD1) [39], again an enzyme that is able to modulate the redox status of the cell [40]. TXNRD1 is overexpressed in certain types of cancer and TXNRD1 expression levels correlate negatively with prognosis [[41], [42], [43]]. Finally, inhibition of TXNRD1 was shown to inhibit tumor growth in a xenograft model [44]. However, development of cancer is not only a cell-autonomous process, but also modulated by the microenvironment and the response of the immune system. Hence, it remained difficult to pinpoint the one selenium-dependent process responsible for tumor formation.

A more recent focus has been on ferroptosis — an iron-dependent, lipid peroxidation–driven form of cell death (Fig. 1C) [[45], [46], [47]]. GPX4, a selenoprotein, is central to suppressing ferroptosis. The enzyme was first purified as a novel liver enzyme reducing lipid hydroperoxides in a glutathione-dependent manner [48]. Tumors often evade ferroptosis by upregulating GPX4, the Sec incorporation machinery, or the cystine/glutamate antiporter system xCT (SLC7A11/SLC3A2) that is needed to increase cellular GSH levels. Several therapy-resistant cancers are sensitive to inhibition of the xCT or GPX4 [49]. Lipid-soluble radical quenching compounds like vitamin E, but also vitamin K or ubiquinone, can synergize with GPX4 in suppressing ferroptosis [[50], [51], [52]]. This mechanism neatly explains why certain forms of tissue damage only occur if GPX4 and vitamin E are deficient at the same time – as in the “dietary liver degeneration” model of Klaus Schwarz [53] which was replicated in liver-specific GPX4-deficient mice fed a low vitamin E diet [50]. In this mechanism, all three “factors” counteracting “dietary liver degeneration” come together: sulfur-containing amino acids as precursors for the co-factor GSH, vitamin E as the other lipid-soluble lipid-hydroperoxide- scavenging compound, and selenium as part of GPX4 the by far most potent lipid-hydroperoxide- degrading enzyme (Fig. 1C).

GPX4 is also needed to sustain T-cell activation. *Gpx4*-deficient T-cells failed to mount antiviral or antiparasitic responses, which could be rescued with pharmacological doses of vitamin E [54].

### 1.5 Spermiogenesis and fertility

Severe selenium-deficiency has long been associated with male infertility, and this connection is now well understood at the molecular level. The selenoenzyme GPX4 serves a unique structural role in spermatozoa. In the midpiece of the sperm, GPX4 becomes extensively cross-linked through its own oxidase activity, stabilizing the mitochondrial sheath [55,56]. Disruption of selenium transport to the testes, either by deleting the plasma selenoprotein SELENOP or its endocytic receptor LRP8, results in the characteristic “kinked sperm” phenotype and male infertility in mice [[57], [58], [59]]. A similar phenotype is observed in mice carrying a heterozygous Sec46Ser mutation in GPX4, which specifically disrupts the mitochondrial isoform [60].

More recently, another sperm-enriched selenoprotein, TXNRD3, has been shown to be essential for normal sperm motility and fertilization. Loss of TXNRD3 in mice not only reduces sperm swimming velocity and fertilization rates but also produces bending defects in the sperm midpiece [61,62].

### 1.6 Brain development and degeneration

Early case reports linked low plasma selenium or reduced GPX activity to intractable childhood seizures [63,64]. Before, neurological decline has been noted in patients rendered Se-deficient during total parenteral nutrition [65]. However, without a concept of how selenium enters the brain and what its roles in the brain might be, these observations remained puzzling [66].

A breakthrough came with the discovery that *Selenop*-knockout mice exhibit progressive movement disorders and epileptic seizures associated with sharply reduced brain selenium levels [[67], [68], [69], [70], [71]]. This and subsequent work clarified that LRP8 and LRP2 receptors mediate SELENOP uptake across the blood–brain barrier [72,73].

Neuron-specific knockouts of either selenoprotein biosynthesis factors [[74], [75], [76]] or specific selenoproteins [46,76] replicated the Se-deficiency phenotype seen in *Selenop*-null mice and revealed that parvalbumin-positive GABAergic interneurons (PVALB^+^ cells) are particularly sensitive to selenoprotein loss [[77], [78], [79]]. Dysfunction of these inhibitory neurons is compatible with the seizure phenotypes observed, and the deficits arise from cell-autonomous processes within the brain rather than being secondary to peripheral disease [80]. Loss of PVALB^+^ neurons was always accompanied by astrogliosis in these models. Strikingly, a naturally occurring deletion of the *SELENOP* gene in dogs causes a near-identical neurological syndrome, including cerebellar ataxia and brain atrophy, confirming that these mechanisms are conserved across mammals [81].

## 2 Brief summary of selenoprotein biosynthesis and metabolism

### 2.1 The canonical selenoprotein biosynthesis pathway

**Fig. 2:**
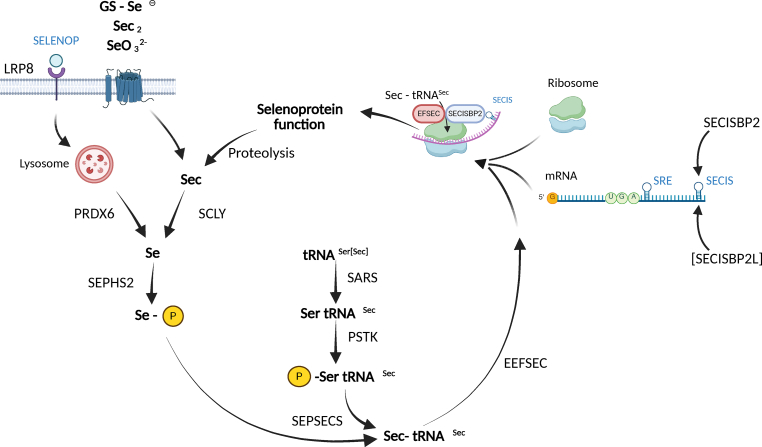
Selenoprotein biosynthesis pathway. Selenium is taken up in different molecular forms through different mechanisms, with peroxiredoxin 6 (PRDX6) as an interactor with selenophosphate synthase 2 (SEPHS2) being the latest addition to the pathway. SEPHS2 generates selenophosphate, the substrate used by selenocysteine synthase (SEPSECS) to convert phosphoseryl-tRNA^Ser[Sec]^ into Sec-tRNA^Ser[Sec]^. Transfer RNA Sec is aminoacylated with Ser by seryl-tRNA synthetase (SARS) followed by phosphorylation through phosphoseryl-tRNA kinase (PSTK). Sec-tRNA^Ser[Sec]^ is bound by elongation factor Sec (EFSEC) to allow translation of a UGA/Sec codon in selenoprotein mRNAs. Selenoprotein mRNAs are characterized by the selenocysteine insertion sequence (SECIS) element in the 3′-untranslated region, which is recognized by SECIS-binding protein 2 (SECISBP2). Some selenoprotein mRNAs also contain the selenocysteine redefinition element (SRE) closely 3′ to the UGA/Sec codon, which can also enhance translation of the UGA codon as Sec. Selenium can be mobilized from Sec by selenocysteine lyase (SCLY). Some SECIS elements are bound by SECISBP2L protein.

Selenoproteins are defined by their incorporation of the rare amino acid selenocysteine (Sec) during translation [82]. Structurally, Sec resembles cysteine, except that the sulfur atom is replaced by selenium. In eukaryotes, Sec is co-translationally inserted at specific UGA codons, which normally signal translation termination. This process requires a dedicated tRNA (tRNA^[Ser]Sec^) and a set of specialized translation factors [83]. A summary of our current understanding of the selenoprotein biosynthesis pathway is summarized in Fig. 2.

A key mechanistic insight is that Sec is normally not present as a free amino acid in the cytosol; instead, it is synthesized directly on its cognate tRNA^[Ser]Sec^: Transfer RNA^[Ser]Sec^ is aminoacylated with serine by seryl-tRNA synthetase (SARS) due to its structural similarity to canonical serine tRNA [84]. Next, the bound serine is phosphorylated by O-phosphoseryl-tRNA kinase (PSTK), yielding phosphoserine (pSer)-tRNA^[Ser]Sec^ [85]. For the next step, selenophosphate is needed. Selenophosphate synthetase 2 (SEPHS2), itself a selenoprotein, generates selenophosphate from selenide and ATP [22], which is then used by selenocysteine synthase (SEPSECS) to convert phosphoserine on the tRNA into Sec yielding Sec-tRNA^[Ser]Sec^ [86,87]. Because free selenocysteine is chemically reactive and potentially toxic due to its high nucleophilicity, this “on-tRNA” synthesis ensures immediate utilization in translation and minimizes the pool of free cytoplasmic Sec.

Despite its interaction with SARS, tRNA^[Ser]Sec^ is structurally distinct from canonical tRNAs [88]. It does not interact with the general eukaryotic elongation factor eEF1A, but instead requires the dedicated elongation factor EFSEC (encoded by *EEFSEC*) [89].

A fundamental question is how the ribosome discriminates between a UGA/Sec codon and the far more common UGA/stop codon, which signals termination in roughly half of all proteins in mammals. In eukaryotes, this is achieved through a selenocysteine insertion sequence (SECIS) element, a stem–loop RNA structure in the 3′-untranslated region of selenoprotein mRNAs [90]. All 25 known human selenoprotein genes are characterized by a SECIS element [91].

The SECIS element is recognized and bound by SECIS-binding protein 2 (SECISBP2) [92], which bridges the ribosome and EFSEC, thereby enabling the insertion of Sec at the UGA codon [93]. Without SECISBP2, selenoprotein synthesis is inefficient, although some residual expression of certain selenoproteins persists in hepatocytes of conditional *Secisbp2*-knockout mice [94].

### 2.2 Mechanisms of selenium Transport and Metabolism

**Fig. 3:**
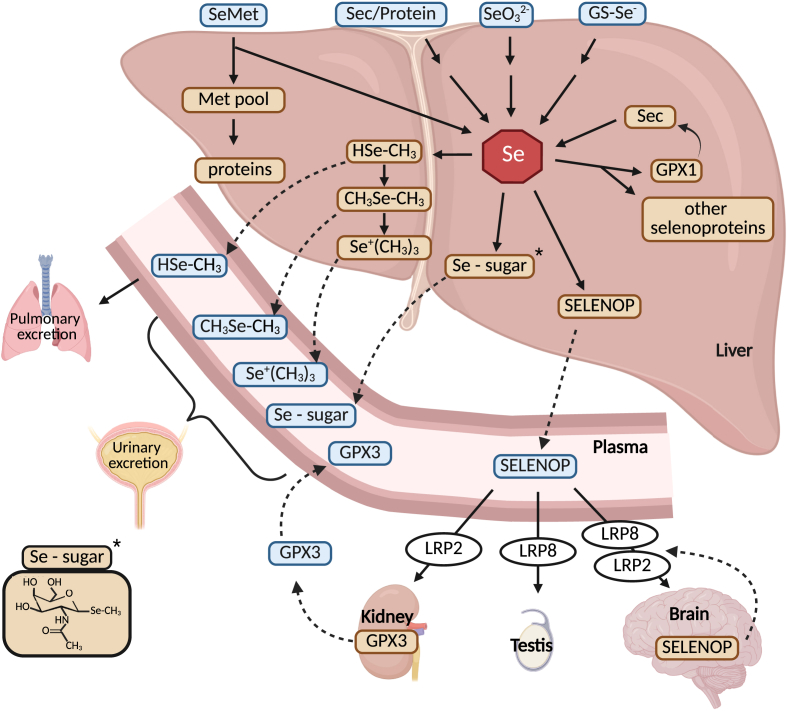
Central role of the liver in selenium metabolism, excretion, and distribution in the body. The liver takes up selenium in inorganic or organic forms, as selenoamino acids or proteins containing selenoamino acids. These are metabolized and used for the biosynthesis of selenoproteins, selenosugars, or methylated selenium compounds. Methylated selenium species and selenosugars are released into the blood stream and excreted via the breath (H_3_CSeH) or urine. Selenoprotein P (SELENOP) distributes selenium in the body and its uptake is mediated by endocytic receptors. The kidney produces plasma GPX (GPX3) as the second abundant plasma selenoprotein. Organs can locally produce SELENOP and directly take it up again in the “SELENOP cycle” which effectively traps selenium in the compartment (e.g. within the brain).

The liver plays a central role in whole-body selenium distribution and homeostasis (Fig. 3). One of its key functions is the secretion of selenoprotein P (SELENOP) into the plasma. SELENOP is unique in carrying up to 12 selenocysteine residues in human, allowing it to function both as a selenium transport protein and a storage form of selenium [[95], [96], [97]]. SELENOP is taken up by cells via endocytic receptors belonging to the LDL receptor-related protein (LRP) family, most prominently LRP8 (also known as ApoER2) and LRP2 (megalin) [58,73]. Receptor expression patterns create a hierarchy of selenium delivery — for example, expression of the high-affinity receptor, LRP8, ensures preferential selenium allocation to the brain and testes, which can maintain their selenium content for some time even under the condition of dietary selenium restriction. The liver can accumulate large amounts of GPX1, possibly more than needed for peroxidase activity. It was speculated that GPX1 serves as a safe, readily mobilizable storage pool of selenium. Recently, it was found that selenosugars, free and in protein-bound form, may account for the rest of bioavailable selenium in the liver [98]. This reservoir may buffer the organism against fluctuations in dietary selenium intake. Selenosugars like 1β-methylseleno-N-acetyl-d-galactosamine, are excreted into urine and may have additional, as yet incompletely understood, biological functions [[99], [100], [101]]. When selenium supply exceeds metabolic needs, selenium is progressively methylated. Mono- and Dimethylselenide can be exhaled trough the lungs, while trimethylselenonium is excreted in the urine. Progressive methylation of excess selenium is mediated by thiopurine-S-methyltransferase (TSMT) and indolethylamine-N-methyltransferase (INMT) [102]. Polymorphisms in INMT have been reported to change trimethylselenonium excretion in human individuals [103]. Known polymorphisms that modulate TSMT activity may likewise affect selenium metabolism.

Some organs, notably the brain, produce SELENOP locally, secrete it into the interstitial space, and then reabsorb it through LRP8-mediated endocytosis. This “SELENOP cycle” helps trap selenium in critical tissues, making them less dependent on daily dietary selenium intake [59,82,96,104].

For new selenoprotein synthesis, selenium can be recycled from proteolytically degraded selenoproteins such as SELENOP or GPX1. Selenocysteine lyase (SCLY) catalyzes the release of selenide from Sec, providing the substrate for selenophosphate synthesis [105].

Selenium can also enter the selenoprotein biosynthesis pathway via selenomethionine (SeMet), which constitutes a small fraction of the total methionine pool in plants and selenium-enriched yeast. Through the *trans*-sulfuration pathway, SeMet can be converted to selenocysteine, because the associated enzymes do not discriminate against SeMet. Dietary selenium supplements often provide SeMet (from yeast) or inorganic forms such as selenite (SeO_3_^2−^) or selenate (SeO_4_^2−^), all of which are highly bioavailable. Inorganic selenium is thought to be reduced in the presence of glutathione (GSH) to form selenoglutathione (GS-SeH) and selenodiglutathione (GS–Se–SG). Selenite and GS-Se-SG can be reduced to selenide by the TXNRD1 system [106].

## 3 Inborn errors of selenoproteins and selenoprotein metabolism in humans

Genetic defects in either selenoprotein-encoding genes or in factors required for their biosynthesis can lead to disorders that overlap with symptoms of dietary selenium deficiency although selenium intake may be normal (Table 1). A comprehensive collection of all genetic variants in ClinVar and gnomAD, also including unpublished variants, is available elsewhere [121].

**Table 1:** Biallelic pathogenic variants in selenoproteins and selenoprotein biosynthesis factors associated with clinical symptoms.

Gene	mutations	activity	Disorder/phenotype	reference
*SELENON*	Missense, frame-shift, non-sense, SRE	Ca^2+^ sensor and redox regulator in ER	Rigid spine muscular dystrophy MIM: 602771	[16]
*SELENON*	Missense, frame-shift, non-sense UGA/Sec Several mutations identical to those in [16]		Multi-minicore disease	[15]
*SELENON*	Deletion, other mutations overlap with [15]		Desmin-related myopathy with Mallory bodies	[107]
*SELENON*	Missense, start codon, mutations overlap with those in [15]		Congenital fiber-type disproportion and insulin resistance	[108]
*SELENON*	SRE, several mutations		*SELENON*-related myopathy	[18]
	
*TXNRD2*	Heterozygous missense∗	Reduction of thioredoxin in mitochondria	Dilated cardiomyopathy	[109]
*TXNRD2*	Non-sense		Familial glucocorticoid deficiency, MIM: 617825, no cardiac phenotype	[110]
*TXNRD2*	missense		Familial glucocorticoid deficiency, micropenis	[111]
*TXNRD2*	missense		Familial glucocorticoid deficiency, micropenis, optic neuropathy, and spasticity	[112]
*TXNRD2*	missense		Familial glucocorticoid deficiency, hyperpigmentation, heart	[113]
*TXNRD1*	missense	Reduction of thioredoxin in cytoplasm	Generalized epilepsy, MIM: 601112	[114]

*GPX4*	Splicing, non-sense	Reduction of lipid-hydroperoxides	Sedaghatian-type spondylometaphyseal dysplasia (SSDM), MIM: 250220	[115]
*GPX4*	Frame-shift, deletion		SSDM	[116]
*GPX4*	missense		SSDM	[117]

*EPT1/SELENOI*	missense	Ethanolamine phosphotransferase/Kennedy pathway to phosphatidyl-ethanolamine and plasmenyl-ethanolamine	complicated hereditary spastic paraplegia (cHSP) MIM: 618768	[118]
*EPT1/SELENOI*	Exon skipping		cHSP, sensorineural deafness, and blindness	[119]
*EPT1/SELENOI*	missense		Seizures, microcephaly, cerebellar atrophy, hypomyelination	[120]
				
Null mutations in selenoprotein genes NOT associated with phenotypes				
*SELENOO*				[121]
*SELENOV*				[121]

Selenoprotein biosynthesis factors				
*SECISBP2*	Missense, non-sense, splice-site	Binding of SECIS element and ribosome	Atypical thyroid hormone resistance, delayed growth, impaired activities of deiodinases; MIM: 609698	[122]
*SECISBP2*	Non-sense		Atypical thyroid hormone resistance	[123]
*SECISBP2*	Non-sense		Atypical thyroid hormone resistance, delayed bone maturation, myopathy, impaired mental and motor coordination development	[124]
*SECISBP2*	Missense, missense disrupting SECIS binding		Atypical thyroid hormone resistance, delayed growth, hearing impairment, UV sensitivity, impaired immune function, azoospermia, axial muscle weakness	[125]
*SECISBP2*	Non-sense, frame-shift		Atypical thyroid hormone resistance, delayed growth, treatment with T3 and GH	[126]
*SECISBP2*	Frame-shift		Atypical thyroid hormone resistance, delayed growth	[127]
*SECISBP2*	Missense, non-sense, frame-shift		Atypical thyroid hormone resistance, delayed growth	[128]
*SECISBP2*	Missense, non-sense, frame-shift, deletion		Atypical thyroid hormone resistance, delayed growth, aortic aneurism	[129]
*SECISBP2*	Missense, non-sense, frame-shift		Atypical thyroid hormone resistance, delayed growth, hearing impairment, absent speech, autistic features, and seizures	[130]

*TRU-TCA1-1* (tRNA^Sec^)	Base change C65G	tRNA^Sec^	Atypical thyroid hormone resistance, delayed growth	[131]
*TRU-TCA1-1* (tRNA^Sec^)	Base change C65G		Atypical thyroid hormone resistance, delayed growth	[132]

*SEPSECS*	missense	Sec biosynthesis from pSer-tRNA^Sec^	Progressive cerebello-cerebral atrophy; ponto-cerebellar hypoplasia 2D; seizures, gray matter loss, infants affected, MIM: 613811	[133]
*SEPSECS*	missense		ponto-cerebellar hypoplasia 2D	[134]
*SEPSECS*	Missense, non-sense		Cerebello-cerebral atrophy, seizures, severe spasticity, and axonal neuropathy, elevated lactate, infants affected	[135]
*SEPSECS*	Missense, splice-site		Hypotonia, global developmental delay, microcephaly, progressive cerebellar atrophy	[136]
*SEPSECS*	Missense, same codon as in [133]		Cerebello-cerebral atrophy, seizures, severe spasticity, and axonal neuropathy, elevated lactate	[137]
*SEPSECS*	Missense, frame-shift		Milder, mental retardation, intellectual disability, late-onset	[138]
*SEPSECS*	Missense		early onset epileptic encephalopathies with burst suppression, dysmyelination, pontine hypoplasia	[139]
*SEPSECS*	missense		slowly progressive cerebellar ataxia and cognitive impairment, late-onset	[140]
*SEPSECS*				[141]
*SEPSECS*	missense		early-onset pyramidal syndrome with optic nerve hypoplasia, later extrapyramidal syndrome featuring dystonia, late-onset	[142]
*SEPSECS*	Missense, splicing		severe global developmental delay, myogenic changes in the lower limbs, but without progressive microcephaly and brain atrophy, late-onset	[143]
*SEPSECS*	Initiation codon, synonymous change affecting splicing		hypotonia, profound developmental delays, and seizures, intellectual disability, reduced mitochondrial activity	[144]
*SEPSECS*	Missense, non-sense		cerebellar atrophy, bradykinesia, ataxia, dystonia, peripheral demyelination, late-onset	[145]
*SEPSECS*	Missense, exon 1 skipping		severe spastic tetraparesis, convergent strabismus and postnatal onset of microcephaly, as well as recurrent blood lactate elevation	[146]
*SEPSECS*	Missense, nonsense, splicing		13 new cases, broad spectrum from mild to severe	[147]

EEFSEC	Missense, initiation codon, frame-shift	Translation factor Sec	global developmental delay, progressive spasticity, ataxia, and seizures, early-onset; MIM: 607695	[148]

Biallelic pathogenic variants in selenoproteins and selenoprotein biosynthesis factors associated with clinical symptoms.

### 3.1 Genetic variation in selenoproteins

The first pathological variants in a selenoprotein gene to be identified were loss-of-function mutations in *SELENON* [16], The disorder is now called SELENON-related myopathy, because several clinical entities turned out to be caused by mutations in SELENON – often the same mutations even produced different clinical presentations [15,16,107,108]. Of particular interest is the finding of several mutations in the so-called selenocysteine-redefinition element (SRE), an RNA hairpin structure immediately 3’ from the UGA/Sec codon in the *SELENON* mRNA [18]. It appears as if the SRE resembles the bacterial SECIS element, which also immediately follows the UGA/Sec codon [149]. The disease has been successfully modelled in transgenic mice and zebrafish [[150], [151], [152]], confirming the essential role of SELENON in skeletal muscle [153]. The mechanism may be that SELENON is a Ca^2+^ sensor in the ER lumen that regulates SERCA pumps [154,155]. However, SELENON-related myopathy does not cause Keshan disease, so either there is a confounding factor exacerbating the disease or another selenoprotein is related to the cardiomyopathy.

Thioredoxin reductase 2 (TXNRD2) is generally considered a mitochondrial enzyme reducing mitochondrial TXN2. Individuals with heterozygous mutations in *TXNRD2* were associated with dilated cardiomyopathy, in line with studies showing cardiac failure in heart-specific *Txnrd2* knockout mice [109,156]. In contrast, mutations, including homozygous prematurely truncating mutations that should completely inactivate the enzyme, were repeatedly reported to cause familial glucocorticoid deficiency, but dilated cardiomyopathy was not reported in these patients [[110], [111], [112], [113]]. Only in one patient a long QT syndrome was diagnosed leaving it open whether some TXNRD2-deficient patients are protected by a genetic modifier or whether the cardiac phenotype is the result of an independent mutation [113]. Studies in adrenal cells showed that TXNRD2 is needed for adrenal (and possibly testicular) steroid biosynthesis and mitochondrial redox balance [112]. TXNRD2 reduces PRDX3 through TNX2 in order to protect mitochondria from reactive oxygen species in adrenal cells. Adrenal steroid producing cells are densely packed with smooth endoplasmic reticulum and many enzymes in the steroidogenic pathway are P450 oxidoreductases, which are notorious for their electron leaks, in particular CYP11B1 [157,158]. This may explain while steroid producing cells are particularly sensitive to loss of TXNRD2 function.

Pathogenic mutations in TXNRD1 have been described in patients with generalized epilepsy [114], consistent with earlier associations between low selenium status and seizure susceptibility. Although complete *Txnrd1* knockout in mice is lethal, neuron-specific deletion of *Txnrd1* affected cerebellar development without producing spontaneous seizures [159].

Loss-of-function variants in *GPX4* result in a particularly severe phenotype disorder that was initially described as Sedagathian-type spondylometaphyseal dysplasia (SSMD), a disease that involves skeletal abnormalities and cardio-respiratory failure in the perinatal period [[115], [116], [117]]. Phenotypic variability may reflect both the nature of the mutation and the activity of compensatory redox systems such as vitamin E, ubiquinone, or vitamin K. In mice, homozygous *Gpx4* deletion is embryonically lethal, and inducible tissue-specific knockouts trigger ferroptosis in affected organs [46,160,161].

Mutations in ethanolamine-phosphotransferase 1 (EPT1, also known as SELENOI) impair the biosynthesis of phosphatidylethanolamines (PE, diacyl-PE) and plasmenyl-PE, causing a severe complicated spastic paraplegia [[118], [119], [120]]. PE is enriched in the brain, and its biosynthesis involves the coupling of DAG to CDP-ethanolamine by EPT1 or CEPT1 (choline/ethanolamine-phosphotransferase). Both enzymes are located in different cellular compartments and CEPT1 cannot compensate fully for the loss of EPT1/SELENOI. Plasmenyl-PE is particularly important in myelin, where it constitutes the majority of PE. Myelination is a late event in development possibly explaining why the mutations in EPT1 lead to a phenotype that develops later than in GPX4-deficiency. PE biosynthesis is obviously of particular importance in the nervous system and spasticity and myelination defects are correlated with EPT1/SELENOI-deficiency while cardio-respiratory symptoms are not reported.

Beyond these examples, homozygous loss-of-function alleles have been identified in genomic datasets for SELENOV and SELENOO, although associated phenotypes remain unreported and thus may not be evident [162].

Variants in other selenoproteins have been linked to disease risk rather than fully penetrant Mendelian syndromes. A systematic review on this subject has been recently published [163]. Here, we highlight just 4 examples in the context of key concepts in this review.

Selenoprotein S was initially cloned as VIMP, a component of the retro-translocon complex that extracts proteins from the ER membrane and targets them for degradation [164]. A promoter polymorphism (−105 G- > A) reducing *SELENOS* expression was shown to correlate with increased release of proinflammatory cytokines [165]. At present there is uncertainty whether *SELENOS* is associated with inflammatory conditions [166], spontaneous preterm birth [167], ischemic stroke [168], or not [169].

The GPX1 p.Leu98Lys substitution has been associated with altered cancer susceptibility in many studies, although not all studies reported significant correlations [163].

The common polymorphism (rs 225014) in *DIO2* denotes a p.Thr92Ala mutation, which leads to lower DIO2 activity in carriers homozygous for the Ala variant [170]. The mutation does not alter the K_M_ of the enzyme and may rather affect the amount of enzyme, because it is located in a peptide associated with DIO2 degradation. In general, the Ala variant has been associated with changes in thyroid function tests which might explain associations with cognitive impairment, hypertension, insulin resistance, and lower psychological well-being [171,172]. However, even in an iodine-deficient region of China, the rs 225014 polymorphism was not associated with mental retardation, while other SNPs in *DIO2* were [173]. A humanized mouse model expressing the Ala92 variant showed decreased DIO2 activity and T3 levels in the brain, and showed ER-stress, although these transgenic mice expressed a His- and YFP-tagged engineered protein [174].

The rare DIO1 mutations p.Asn95Lys and p.Met201Ile are associated with reduced enzymatic activity and thus lead to elevated rT3 and abnormal rT3/T3 ratios, although no immediate clinical phenotype was reported [175].

### 3.2 Genetic variation in biosynthetic machinery

A milestone in the genetics of selenoprotein biosynthesis was the identification of patients carrying mutations in *SECISBP2* [122]. Quite unexpectedly at the time, these patients were diagnosed because of delayed growth and bone age and it was found that the phenotype is caused by reduced activities of deiodinases. Considering extended thyroid function tests, the data imply that (hepatic) DIO1 activity was reduced explaining the increased rT3 and T4 levels. In addition, a blunted response of the pituitary to T4 challenges suggested a reduced activity of DIO2, at least in the pituitary [122]. Plasma selenoproteins GPX3 and SELENOP were reduced [122]. From then on, more patients with this phenotype were discovered, but also several patients which showed additional clinical symptoms. These included myopathy – reminiscent of *SELENON*-related myopathy – hearing impairment, azoospermia, increased UV-sensitivity, and impaired immune function, even impairment of mental and motor coordination development in one patient [124,125]. Hearing impairment may be directly explained by the role of DIO2 in the development of the inner ear, as shown in mice [30]. Non-sense mutations in the N-terminal domain of SECISBP2 do not lead to complete loss-of-function, because initiation at downstream AUG codons is possible and the C-terminal part of the protein seems to harbor all domains needed for SECIS binding and Sec incorporation [123]. T3 and growth hormone treatment was reported to improve growth in one patient [126]. The same patient was treated with α-tocopherol, a treatment that improved lipid peroxidation markers and increased white blood cell counts [176]. A novel finding is the formation of aortic aneurisms in a fraction of patients carrying SECISBP2 mutations [129]. Currently it appears that in patients a moderate impairment of selenoprotein expression is revealed by changes in the thyroid hormone axis and delayed growth and bone maturation. In more severe cases, myopathy and mild hearing impairment come on top of these symptoms, and only the most severe mutations lead to neurodevelopmental defects [130]. Among the neurological findings were frequently motor and intellectual disability, and some showed seizures. Some of these patients were treated with T3 to prevent developmental delay. Studies in mice with neuron-specific *Secisbp2* genetic inactivation showed that the expression of selenoproteins was only partially reduced in the brain, but resulted in seizures, dystonia, and coordination defects [74,94,177,178]. Thus, the mild phenotype of the initially reported SECISBP2 patients cannot simply be explained by compensation through SECISBP2L (see below, chapter 4).

Very rare patients with mutations in the tRNA^[Ser]Sec^ gene (human gene symbol: *TRU-TCA1-1*) largely phenocopy the thyroid hormone-related phenotypes of the classical form of *SECISBP2*-deficiency [131,132]. It was speculated that the base-change affected post-transcriptional modification of the tRNA^[Ser]Sec^ [131], but it is also possible that this tRNA^[Ser]Sec^ is less stable or does not efficiently interact with enzymes. Vindry et al. recently demonstrated that the mutated tRNA^[Ser]Sec^ disrupts the hierarchical expression of selenoproteins [179].

Since Sec is invariably formed on the tRNA^[Ser]Sec^ from pSer-tRNA^[Ser]Sec^, the enzyme performing this step, SEPSECS, is essential (Fig. 2). Mutations in *SEPSECS* were identified in patients with progressive cerebello-cerebral atrophy, an early-onset neurodegenerative disorder [133,135]. These patients suffer from seizures, cerebral atrophy, myelination defects, and cerebellar hypoplasia, all phenotypes described in selenoprotein-deficient mice before (Table 1). Later, when more patients with *SEPSECS* mutations were diagnosed, cases with late-onset and slower progression of the disease were found [138,140]. Taken together, a recent study with 27 affected individuals reported three general clinical courses, i) early-onset with severe cerebral atrophy, ii) milder early-onset with milder deterioration, and iii) late-onset with milder progression [147]. The phenotypes and age of onset in patients affected with *SEPSECS* mutations seem to correlate with the severity of mutations or residual enzymatic activity. Seizures occur in those with early onset, while dystonia and demyelination are features at late onset. Quite remarkably, when thyroid function tests were reported, T4 was not found abnormal as in *SECISBP2*-deficiency. When myopathy was reported, it was associated with mitochondrial impairment and elevated lactate (Table 1).

Very recently, several pedigrees with members carrying pathogenic variants in *EEFSEC* have been described [148]. These patients, display neurological phenotypes covering a spectrum of severity similar to patients with *SEPSECS* mutations. Again, the phenotype is clearly neurological with no involvement of the thyroid axis reported. This discrepancy of phenotypes related to thyroid hormones in contrast to severe neurological phenotypes may be explained by a more severe disruption of selenoprotein translation, if the co-translational incorporation of Sec-tRNA^[Ser]Sec^ is impaired as opposed to the impaired recruitment of selenoprotein mRNA to the ribosome.

Either some selenoproteins are capable of being translated at a minimum level without the help of a SECIS-binding protein (as shown in mice, [74,94,177]) or SECISBP2L can compensate in some cell types for impaired SECISBP2 activity in translation of the more important selenoproteins.

### 3.3 Missing pieces

To date, mutations in two essential selenoprotein biosynthesis factors have not been published in humans or in mouse models: SEPHS2, a selenoprotein itself, and PSTK (Fig. 2). If one dared to predict, patients affected with pathological variants in these genes will likely resemble patients with mutations in *SEPSECS* or *EEFSEC*. In addition, more severe mutations in tRNA^[Ser]Sec^ might also lead to a neurological phenotype considering the mouse model with a hypomorphic promoter mutation in the tRNA^[Ser]Sec^ gene [79].

Milder phenotypes might be expected from patients with inefficient Se/Sec recycling. Their phenotypes will likely depend on dietary selenium intake – and should favorably respond to dietary selenium supplementation. This prediction rests on the phenotypes of knockout mice for *Scly*, *Selenop*, and *Lrp8* [77,78,[180], [181], [182]].

With regards to individual selenoproteins, there is a remarkable discrepancy between mice and men [162]: While all three deiodinase genes have been inactivated in mice and all homozygous mutant mice were viable [[183], [184], [185]], there is no report on patients carrying homozygous null alleles in *DIO1*, *DIO2*, or *DIO3*. Clearly, deiodinases are involved in developmental decisions in many organs [186], but beyond the relatively mild phenotypes of individuals carrying the p.Ala92Thr mutation in *DIO2* or the two rare *DIO1* mutations described above, there is a conspicuous paucity of reports from humans. *DIO3* mutations may be more severe, as seen in *Dio3*^*−/−*^ mice, and imprinting of the gene may already affect heterozygous carriers in a tissue-dependent fashion [183,187].

### 3.4 Difficult cases

SEPHS1 was initially believed to be the eukaryotic homolog of the bacterial SelD/selenophosphate synthase, although this activity was not unequivocally demonstrated [188]. When other selenophosphate synthases were cloned, also from archaea and bacteria, it became apparent that SEPHS1 has a Thr at the position where active selenophosphate synthases have the catalytic Sec or Cys. Why knockout of *Sephs1* affected only two selenoproteins in mouse liver remains unclear [189], but potentially it is involved in redox regulation and may affect selenium recycling from Sec [190,191]. This gene, however, has not come up in those genetic screens that found all other selenoprotein biosynthesis factors and even revealed peroxiredoxin 6 (PRDX6) as a new gene involved in the pathway [[192], [193], [194]] (see below, chapter 4).

Secp43 (now designated TRNAU-AP1) was shown to associate with tRNA^[Ser]Sec^ and its knock-down affected selenoprotein biosynthesis in cultured cells [195,196]. However, conditional gene inactivation in mice failed to demonstrate a role in selenoprotein biosynthesis, at least in hepatocytes and in neurons of selenium-replete animals [197]. Again, none of the recent genetic screens in cells that identified *PRDX6*, reported *TRNAU-AP1* as a hit [[192], [193], [194]].

SELENBP1 was described as a selenium-binding protein, but is not a selenoprotein and does not require selenium for catalysis [198,199]. Whether it has any clearly defined role in selenium metabolism has not been convincingly shown, but it is reported to be induced by selenium supplementation [200]. Its absence from the coessentiality network around GPX4 and the selenoprotein biosynthesis machinery may indicate it is not involved in this process [[192], [193], [194]]. Recently, it was described as a methane-thiol-oxidase involved in the breakdown of methanethiol (CH_3_SH) [201]. It's deficiency in humans or mice leads to increased methanethiol exhalation and a cabbage-like smell. Based on similarity, methylselenol may also be a substrate of SELENBP1.

A number of RNA-binding proteins have been described that interacted with SECIS elements or selenoprotein mRNAs and potentially regulated selenoprotein expression in various assays [[202], [203], [204], [205]]. The authors of this review are not aware of any report that any of these RNA-binding proteins reappeared in genetic screens or were associated with any phenotype related to selenium metabolism in humans. One RNA-binding protein, eIF4a3, was shown to modulate selenoprotein biosynthesis in cells [206] and was recently reported to be up-regulated in prostate cancer where its induction reduced the expression of SELENOF, a selenoprotein whose loss correlated with the transformed phenotype [207].

Thus, it cannot be excluded at this moment, that the genes with weaker evidence are of significance for selenoprotein expression. For example, mice deficient in *Prdx6* and *Ftsj1* have not been challenged with a low selenium diet and thus the contribution of these genes to selenoprotein biosynthesis was not readily revealed (see below, chapter 4A). Also, combination of genetic deficiencies may be important: Mice deficient in *Scly* do not develop a phenotype on selenium-deficient diet, but are more susceptible when combined with *Selenop*-deficiency [181,182]. Since the role of FTSJ1 was revealed only in the very special case of micrometastasis in melanoma, it is possible that any of the “weak cases” above may surface again as a modulator of selenoprotein expression under very specific pathologic conditions, modulated by selenium in the diet, and depend on genetic variation in other loci.

## 4 Emerging aspects in selenium metabolism and action

In the following paragraphs we present recent work that added important new aspects or concepts to selenoprotein biosynthesis or the roles of selenoproteins in health and disease.

### 4.1 Selenoprotein biosynthesis

#### 4.1.1 SECISBP2L: A paralog with specialized roles

A paralog of SECIS-binding protein 2 (SECISBP2L) was identified over a decade ago [208]. Initial studies suggested only a minor contribution to selenoprotein biosynthesis in mammalian cells, at least as long as SECISBP2 was present, while SECISBP2L from an invertebrate that does not have a SECISBP2 gene was able to direct selenoprotein translation [209]. However, residual selenoprotein expression persists after *Secisbp2* deletion in mouse hepatocytes [94]. More recently, zebrafish experiments revealed that in the absence of *sbp2*, a subset of selenoproteins could still be synthesized — a capacity lost in *sbp2/sbp2l* double mutants [210]. The results suggest that zebrafish sbp2l can interact with the SECIS elements of some selenoproteins and facilitate selenoprotein biosynthesis. If sbp2 and sbp2l are regulated differentially, this mechanism may contribute in part to the hierarchy of selenoprotein expression under conditions of selenium deficiency.

While it is still not clear whether SECISBP2L can compensate for loss of SECISBP2 in mammals, recently it was reported that SECISBP2L binds the SECIS elements of *Dio2* and *Dio3* mRNAs [211]. In differentiating oligodendrocytes, SECISBP2L is highly expressed and required for DIO2 expression. The authors showed that conditional *Secisbp2l* knockout mice had reduced conversion of T4 to T3 during myelination leading to hypomyelination, which was rescued by the T3 receptor agonist sobetirome [211]. In contrast, another study showed that in constitutive *Secisbp2l*-knockout mice oligodendrocyte function was disrupted, but the authors attributed the effect to SECISBP2L binding to SECIS-like RNA structures in non-selenoprotein mRNAs and even demonstrated no effect on selenoproteins in these mice [212]. These authors did not specifically study *Dio2* and *Dio3* mRNAs, but they did not find any selenoprotein mRNAs changed in the transcriptome. Therefore, it remains an open question whether, in which organs, and regarding which selenoproteins SECISBP2L is important for the translation of selenoproteins in the absence of SECISBP2.

#### 4.1.2 PRDX6: A link between selenium recycling and ferroptosis defense

A functional connection between selenium utilization and PRDX6 was initially suggested through analysis of a co-essentiality screen [194]. Three independent studies confirmed that PRDX6 participates in delivering selenium to the biosynthesis machinery, particularly under conditions where GPX4 activity is critical for ferroptosis resistance.

Ito et al. showed that PDRX6 would interact with GS-Se-SG and deliver selenium via its Cys47 to the selenoprotein biosynthesis machinery [213]. They confirmed the co-essentiality of PRDX6 with selenoprotein biosynthesis and GPX4, and showed that *Prdx6*-KO mice showed lower selenoprotein expression in the brain, but not in liver and kidney [213]. More precisely, Fujita et al. performed an elegant genetic screen for iron-triggered ferroptosis in mouse fibroblasts rendered iron-sensitive through *Fbxl5*-inactivation [193]. They also identified PRDX6 as a gene involved in provision of selenium to GPX4 and showed that PRDX6 lacking Cys47 was not able to perform this activity [193]. The authors further showed that PRDX6 binds selenide (Se^2−^) through Cys47 and provides the selenide to SEPHS2 through direct interaction. Using a similar genetic assay that probed the sensitivity of cells being selenium-dependent through LRP8-dependent SELENOP uptake, Chen et al. showed that a pathway acting in parallel to selenium liberation by SCLY existed that relies on PRDX6 [192]. These authors demonstrated by mass-spectrometry the binding of Se^2−^ to Cys47 in PRDX6 and also provided evidence for its interaction with SEPHS2 [192]. Finally, they showed that growth of neuroblastoma cells in mice was greatly diminished when both, PRDX6 and SCLY, were inactivated [192]. While these studies, together, place PRDX6 firmly in the selenoprotein biosynthesis pathway, it should be noted that the lack of PRDX6 also impacts on lipid metabolism and may modulate ferroptosis through more than one pathway [214].

#### 4.1.3 FTSJ1: tRNA modification and the hierarchy of selenoprotein expression

**Fig. 4:**
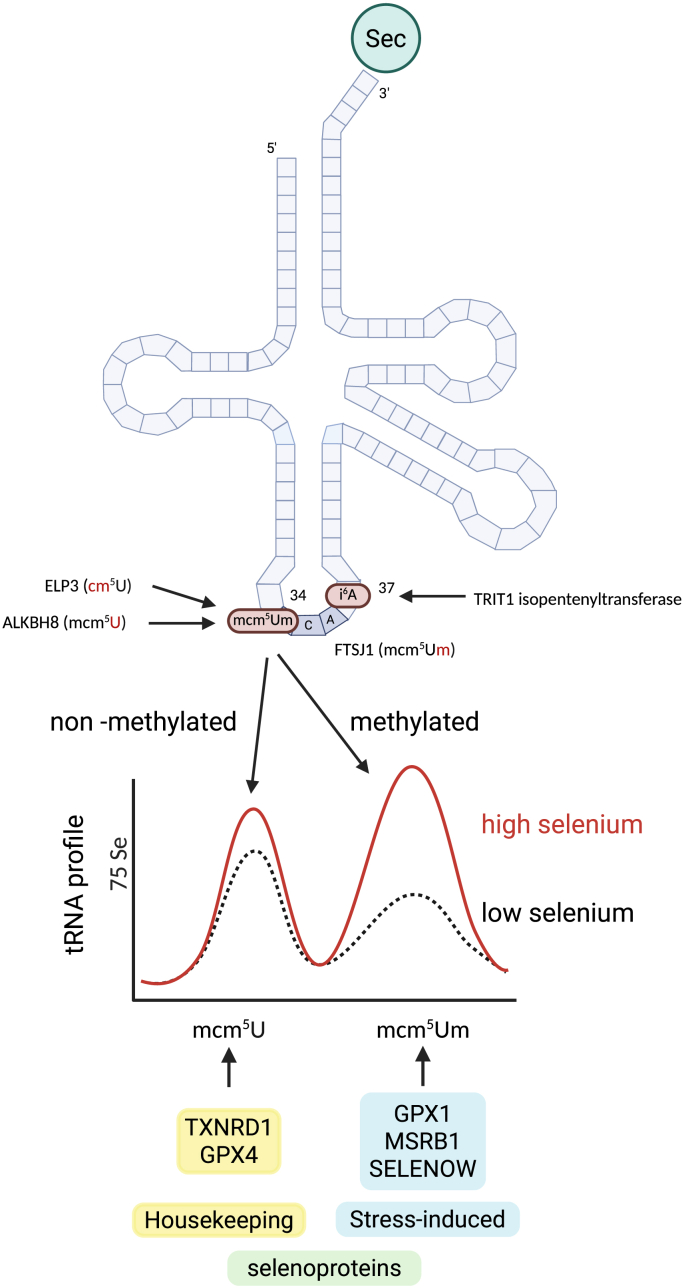
The concept of translational regulation of selenoprotein expression depending on the modification of tRNA^[Ser]Sec^ in the anticodon stem loop. Several enzymes contribute to post-transcriptional modifications of tRNA^[Ser]Sec^. Two species of tRNA^Sec^ can be separated by chromatographic methods, depending on the presence of the 2′-O methyl group Um34. Increased selenium availability leads to a higher proportion of methylated of tRNA^[Ser]Sec^. Essential selenoproteins are readily translated even at low methylated tRNA^[Ser]Sec^ levels, but so-called “stress-induced” selenoproteins are better translated using the methylated tRNA. FTSJ1 is the 2′-O methylase involved.

Transfer RNAs carry many post-transcriptional modifications that modulate their functions [215]. Therefore, to fully characterize tRNA^[Ser]Sec^, it was important to determine its modified bases [216,217]. Mammalian tRNA^[Ser]Sec^ contains several modifications in the anticodon loop that were thought to impact its function: the i^6^A37 modification close to the anticodon, mcm^5^U34 on the base decoding the wobble position, and a 2′-O methylation of ribose 34 (Um34) (Fig. 4). Isopentenylation of A37 depends on TRIT1 [218], but is not required for selenoprotein expression in murine hepatocytes and neurons [219]. The complex mcm^5^U modification of U34 depends on the elongator complex and ALKBH8 [220,221]. Remarkably, methylation of ribose 34 was shown to correlate with selenium availability and seemed to enhance biosynthesis of “stress-related” selenoproteins, namely GPX1 [216,[222], [223], [224]]. The reader is referred to a recent review covering the details of this topic [88]. The differential usage of methylated versus non-methylated tRNA^Sec^ would thus contribute to the observed “hierarchy” among selenoproteins, i.e. some selenoproteins are very sensitive to limited selenium supplies because they rely on the methylated tRNA^[Ser]Sec^ (e.g. GPX1), while others are still being readily made in the presence of only low amounts of selenium that correlates with non-methylated tRNA^[Ser]Sec^, e.g. GPX4 and TXNRD1 (Fig. 4). While the concept seemed straightforward, a clear example where this process would be of biological significance remained elusive until recently.

The methylase responsible for 2′-O methylation at base 34 was identified as FTSJ1. The gene was initially associated with X-linked mental disability [225], and the disease mechanism is likely linked to impaired translation of neuronal proteins, if the 2′-O modification of tRNA^Phe^ is incomplete [226]. Although tRNA^[Ser]Sec^ is also a substate of FTSJ1, *Ftsj1*-knockout mice did not show a defect in selenoprotein expression [219,226]. Thus, it came as a surprise when a recent paper showed that melanoma cells apparently depend on FTSJ1 activity to promote efficient selenoprotein expression during formation of micro metastases [227]. The authors argued that under these specific conditions, single cells and micro metastases are exposed to enhanced oxidative stress and up-regulate several selenoproteins – apparently by inducing FTSJ1 expression [227]. Along these lines, inactivation of *FTSJ1* in melanoma cells rendered them more sensitive to oxidative stress and impaired formation of metastases [227].

### 4.2 Deiodinases in cancer

Deiodinases can regulate cell fate decisions during development, regeneration, and cancer formation [228]. While there is a large literature on correlations of deiodinase expression with different types of cancers, we will focus here on experimental studies mainly using *in vivo* models. In a series of publications, it was shown that DIO3 expression is increased in skin stem cells and basal cell carcinoma, and that reducing DIO3 expression or T3 application can impair tumor growth [229,230]. This led to the concept that DIO3 expression “protects” stem cells – or cancer – from the differentiating action of T3 [231]. DIO3 also contributes to metabolic reprogramming in ovarian cancer, and its inhibition was shown to attenuate tumor growth [[232], [233], [234]]. On the contrary, overexpression of DIO2 was shown in a mouse model of adenomatous polyposis and inhibition of deiodinases suppressed tumor formation [235]. Since then it emerged that tumor invasion profits from increased local T3 levels caused by DIO2 overexpression [236]. Along these lines, loss of DIO1 also promotes ovarian cancer progression [237]. However, here it is unclear whether it is the 5-deiodinase or the 5‘-deiodinase activity of DIO1 that is critical for tumor biology (Fig. 1A). Thus, considerations on selenium and cancer cannot exclusively be seen from the perspective of ferroptosis, but must consider as well deiodinases [238]. Potential deiodinase inhibitors and considerations how to apply them in cancer have recently been published [239].

### 4.3 Selenoproteins in blood and the immune system

Selenoproteins are involved in the regulation of stress erythropoiesis, in particular SELENOW in mice [240]. It is an old observation that tissue damage is greater under conditions of an activated immune system when combined with selenium deficiency. An answer to this question has now been found in a defect of macrophages to switch from the pro-inflammatory M1 phenotype to the anti-inflammatory M2 phenotype. In fact, SELENOW regulates this process and *Selenow*-deficient macrophages are impaired in the resolution of inflammation [241]. Neutrophils are also selenium-dependent and this work has recently been reviewed [242].

Not only inborn immunity, but also adaptive immunity is affected by selenium. As a reminder, SEPHS2 and TXNRD1 have been found as genes induced during T-cell activation [22,39]. Two groups have shown that SELENOI is required for activation of (follicular) helper cells [[243], [244], [245]]. More recently, SELENOK has been described in T-cell activation [245] and B-cell activation [246]. Taken together, this recent and older work supports the notion that a low selenium status impairs both inborn and adaptive immunity. The more recent data now pinpoint which selenoproteins are important for modulating immunity.

### 4.4 New aspects on the function and regulation of selenoproteins

#### 4.4.1 SELENON

Messenger RNA editing emerged as a surprising new mechanism of regulation of *SELENON* expression [247]. The authors demonstrated that ADAR1-dependent mRNA editing affects utilization of a splice site that directs inclusion of an ALU element into the *SELENON* mRNA. As editing is developmentally regulated, formation of functional *SELENON* mRNA is developmentally regulated [247].

The function of SELENON and the role of the Sec within the protein remains enigmatic. It is, however, clear that SELENON-deficiency is associated with ER-stress [154,155]. A recent paper suggests that the ER-stress associated with SELENON-deficiency may be druggable. The authors showed that inactivation of the endoplasmic reticulum oxidase ERO1A partially rescues *Selenon*-deficient mice [248]. However, this does not mean that the role of SELENON is to balance the redox state in the ER. Rather, ERO1A is induced by ER-stress, and accordingly, treatment of *Selenon*-deficient mice or *SELENON*-deficient muscle fibers with the chemical chaperone tauroursodeoxycholic acid (TUDCA) mitigated ER-stress and improved muscle function [248].

#### 4.4.2 Ferroptosis regulation and selenoproteins in metastasis

Some ferroptosis inhibitors reduce the amount of GPX4 expressed [117]. While inhibition of GPX4 was generally believed the mode of action of the ferroptosis inducers RSL3 and ML162, it was shown that these compounds do not directly inhibit recombinant GPX4, but bind to and inhibit TXNRD1 [249]. Still, cell death by other inhibitors of TXNRD1 cannot be suppressed by certain ferroptosis inhibitors, thus it is likely that both systems interact via pathways that are not entirely clear at the moment.

Triple negative breast cancer (TNBC) cells upregulate TXNRD1. Direct inhibition of TXNRD1 by a novel compound attenuated several aggressive cancer phenotypes of TNBC cells and reduced their growth in a mouse model [250].

Modulating selenoprotein biosynthesis in general is another protective mechanism of cancers against ferroptosis. Increased uptake of selenium/SELENOP via LRP8 [251,252] is one way to increase GPX4 expression. In turn, hepatocarcinoma can boost local selenoprotein expression by retaining selenium within the cell by down-regulation of SELENOP, which would otherwise export selenium [253]. The authors of this paper showed that down-regulation of SELENOP was mediated by NRF2, which also up-regulated TXNRD1.

TNBC cells are primed for ferroptosis, but can evade the metabolic cell death, once they have reached a higher cell density. The protective factor was found to be mono-unsaturated fatty acids, which are produced by stearoyl-CoA-desaturase (SCD), an enzyme expressed stronger at higher cell density [254]. Mono-unsaturated lipids are less prone to peroxide propagation than poly-unsaturated lipids. SCD was already identified before as a gene negatively correlated with selenoprotein biosynthesis machinery in co-essentiality analyses, i.e. protective against ferroptosis [194]. Targeting selenoprotein biosynthetic factors, SEPSECS or SEPHS2, in triple-negative breast cancer cells reduced lung metastasis in a xenograft tumor model [254]. This concept nicely aligns with the importance of FTSJ1 during the formation of micro metastases in melanoma [227].

The possibility that selenium may play a particular role in metastasis has been proposed a decade ago [255]. Very recently, a new mechanism was proposed implicating SELENOO in the formation of metastases by mouse melanoma cells [256]. These authors demonstrated that SELENOO AMPylates mitochondrial proteins, e.g. SDHA and ACO2, thereby increasing complex II activity and thus ATP production and NADPH availability. Inactivation of SELENOO in melanoma cells impaired their rate of metastasis formation in a mouse xenograft model, and application of N-acetyl cysteine as an antioxidant compensated for the loss of SELENOO [256]. Thus, taken together two intertwined concepts are emerging: selenoproteins as protectors from cell death and promoters of tumor/metastasis formation, in particular during the vulnerable stage of single cells and micro metastases that have not yet established a safe niche.

On the contrary, drugs that inhibit ferroptosis may also be useful. A promising route towards a therapy in GPX4-deficiency may be small molecule drugs with lipid-hydroperoxidase (GPX4-like) activity, which protected primary neurons from ferroptosis [257]. Of more limited use may be a compound that restores GPX4 activity from a pathogenic GPX4 variant, GPX4^R152H^, and thus represents hope for patients carrying this mutation in homozygosity [258]. There may even be a way to reduce lipid peroxidation via pharmacological doses of selenite without the involvement of selenoproteins: hydrogen selenide produced via selenite reduction was shown to reduce mitochondrial ubiquinone and thus counteract lipid peroxidation [259]. The authors showed that sulfide:quinone oxidoreductase (SQOR) was catalyzing this process and overexpression or knockout of the enzyme accordingly modulated ferroptosis sensitivity of the cells.

#### 4.4.3 Selenium and selenoprotein actions in the brain

SELENOP was identified as an essential factor for the survival of cultured neurons [260]. Its role in distribution of selenium to organs and also to single cells was discussed above (Fig. 3). Here, we point out the very specific function of the SELENOP/LRP8 pair for the stimulation of neuronal precursor cells in the dentate gyrus. An elegant study delineated that the well-described effect on hippocampal neurogenesis by physical activity correlated with the increased release of SELENOP into plasma and could be stimulated by cerebral infusion of inorganic selenium [261]. The authors further showed that both, genetic inactivation of *Selenop* or *Lrp8*, blunted or abolished the increase of neurogenesis in the dentate gyrus. Finally, they suggested that the age-related decline in hippocampal neurogenesis and performance in spatial navigation tasks could be partially rescued by dietary selenium supplementation [261]. These findings lend further support to a study that correlated increased cognitive decline in the elderly with decreasing plasma selenium levels [262].

Mouse models deficient in selenoproteins or GPX4 alone exhibit a reduced number of PVALB^+^ GABAergic interneurons in the cerebral cortex [74,75,[77], [78], [79]]. A recent study investigated the selenium-dependence of cortical neurons *in vitro* and showed that the maturation of PVALB ^+^ cells, i.e. the formation of the functionally important perineuronal nets, correlated with selenium in the medium [263]. The authors further showed that selenium correlated with the number of glutamatergic inputs and with spontaneous network activity. In addition, it was shown that mice raised on a mildly selenium-deficient diet may not display decreased PVALB^+^ neuron numbers, but yet show reduced formation of perineuronal nets around those cells [263].

The phenotypic similarity of patients affected by *SELENOI/EPT1*-deficiency and patients with pathogenic variants in *PCYT2*, another gene involved in plasmalogen biosynthesis, strongly suggests that impaired biosynthesis of plasmenyl-PE is the major underlying reason for the disease [120]. These lipids are abundant in myelin and have been shown to protect nerves from oxidative damage to lipids, possibly because the vinyl ether moiety is preferentially oxidized, but does not propagate the lipid peroxidation [264]. Accordingly, mice conditionally deficient in SELENOI in the nervous system showed a myelination defect and significantly reduced abundances of phosphatidyl-ethanolamine (PE), plasmanyl-PE, and plasmenyl-PE [265]. The corresponding choline-containing lipids, phophatidylcholine (PC), plasmanyl-PC, and plasmenyl-PC lipids were correspondingly increased. In keeping with the notion that plasmenyl-PE can protect from lipid oxidation, lipid peroxidation was increased in oligodendrocytes and oligodendrocyte precursor cells [265]. The mouse model thus supports and complements the observations from patients affected with pathogenic variants in *SELENOI* [[118], [119], [120]]. Whether, however, dysregulation of SELENOI causally contributes to amyotrophic lateral sclerosis in a significant fraction of the cases remains to be corroborated by additional data [266]. Taken together, sensitivity to ferroptosis clearly depends on the metabolic state of the cell and on the availability of antioxidants that modulate lipid peroxidation or on unsaturated lipids and their metabolism. The involvement of SELENOI/EPT1 in plasmalogen metabolism adds a second selenoprotein to this equation. It is interesting that the predominant symptoms associated with deficiency in GPX4 and SELENOI/EPT1 primarily involve the nervous system.

#### 4.4.4 Autoimmunity to SELENOP

Plasma levels of SELENOP correlate with selenium intake and are central to selenium distribution in the body. An emerging new concept is autoimmunity against SELENOP, which antagonizes its function as a selenium carrier and affect selenium-dependent processes in SELENOP-dependent organs. Autoantibodies against SELENOP have been found in patients with several diseases and correlate with adverse outcomes. For example, SELENOP autoantibodies were increased in Hashimoto thyroiditis [267], predicted breast cancer recurrence [268], were about ten-fold more prevalent in patients afflicted with chronic fatigue [269], and predicted colorectal cancer mortality [270]. SELENOP may be particularly prone to autoimmunity, because it is known that of the 10 Sec moieties according to UGA codons in its mRNA, only approximately 5-8 are indeed present [271], while others are replaced by near-cognate amino acids under conditions of selenium-deficiency or aminoglycoside exposure [272]. This means that under the condition of severe burn injury, for example, when selenium levels plunge significantly, SELENOP species may be made that contain peptides not represented in the *SELENOP* gene eliciting an autoimmune response that would later interfere with SELENOP function. In fact, blood samples obtained from individuals with burn injuries at several time points after the incident allowed to substantiate this hypothesis of acquired SELENOP autoimmunity [273].

## 5 Summary

Selenium research mirrors the success of science during the last decades. First, selenium seemed an interesting oddity, but its relevance to human health was difficult to delineate without a thorough understanding of selenium metabolism, selenoprotein biosynthesis, and an idea what these selenium-containing proteins are actually doing. In hindsight, early health claims were not that wrong at all, but studies were often underpowered or anecdotal. Rigorous application of genetics and molecular biology helped to isolate the functions of individual selenoproteins and to elucidate the precise mechanisms in which selenoproteins are involved.

GPX4, to name just one selenoenzyme, is clearly at the center of ferroptosis and associated biology. Other selenoenzymes are finally assigned to biochemical functions or their functions are related to important biological processes.

While these studies are still in progress and flourishing, some old questions that kept lingering around are eventually coming closer to a substantial answer. Does selenium deficiency cause – or exacerbate – disease? And which diseases? Does selenium supplementation improve selenium status and health? And, importantly, what is “selenium status”?

The historical episode about trouble shooting by Klaus Schwarz illustrates an important lesson for modern selenium research. We must not overlook the modulating effects of real-world dietary differences in selenium status — a factor that still influences human health in more than subtle and important ways. Diets are different around the world, not only with respect to selenium content. Obviously, there are interactions of selenium-dependent processes with iodine and vitamin E, possibly co-enzyme Q, sulfur-containing amino acids, and those factors that modulate all of the former. In addition, genetic differences in selenium metabolism, uptake, distribution, and excretion, may modulate how effectively an individual makes use of dietary selenium. Finally, acquired autoimmunity against SELENOP adds another layer of complexity to these processes that is beyond genetics.

Much more research is needed to bring our current biological understanding to fruitful application in the clinics. For example, is it good to supplement a cancer patient with selenium in order to boost the individual's immune system or is additional selenium, on the contrary, rescuing cancer cells from certain death by ferroptosis? Is blockade of certain deiodinases good or bad in other cancers? Will drugs that inhibit TXNRD1 specifically kill cancer cells or will the drugs have too severe unwanted side effects? Is SELENOP merely a prognostic marker at the onset of disease or does improvement of the SELENOP level improve the prognosis of a patient?

## CRediT authorship contribution statement

**Ulrich Schweizer:** Conceptualization, Writing – original draft, Writing – review & editing. **Marietta Fabiano:** Conceptualization, Visualization, Writing – review & editing.

## Declaration of competing interest

None.

## Data Availability

No data was used for the research described in the article.
